# Enhancing European Management of Analgesia, Sedation, and Delirium: A Multinational, Prospective, Interventional Before-After Trial

**DOI:** 10.1007/s12028-023-01837-8

**Published:** 2023-09-11

**Authors:** Nicolas Paul, Julius J. Grunow, Max Rosenthal, Claudia D. Spies, Valerie J. Page, James Hanison, Brijesh Patel, Alex Rosenberg, Rebecca von Haken, Urs Pietsch, Claudia Schrag, Christian Waydhas, Peter Schellongowski, Elisabeth Lobmeyr, Michael Sander, Sophie K. Piper, Daniel Conway, Andreas Totzeck, Björn Weiss

**Affiliations:** 1 Department of Anesthesiology and Intensive Care Medicine (CCM/CVK), Charité – Universitätsmedizin Berlin, Corporate Member of Freie Universität Berlin and Humboldt-Universität zu Berlin, Berlin, Germany; 2 Berlin Institute of Health, Berlin, Germany; 3 Department of Anaesthesia, Watford General Hospital, Watford, Hertfordshire UK; 4 Manchester Royal Infirmary, Manchester University National Health Service Foundation Trust, Manchester, UK; 5 Division of Anaesthetics, Pain Medicine and Intensive Care, Imperial College London, London, UK; 6 Royal Brompton and Harefield National Health Service Foundation Trust, London, UK; 7 Department of Anesthesiology, University Hospital Heidelberg, Heidelberg, Germany; 8 Department of Anesthesiology and Intensive Care Medicine, Kantonsspital St. Gallen, St. Gallen, Switzerland; 9 Clinic of Intensive Care Medicine, Kantonsspital St. Gallen, St. Gallen, Switzerland; 10 Department of General and Trauma Surgery, BG University Hospital Bergmannsheil, Bochum, Germany; 11 Medical Faculty, University Hospital Essen, University of Duisburg-Essen, Essen, Germany; 12 Department of Medicine I, Medical University of Vienna, Vienna, Austria; 13 Department of Anesthesiology, Intensive Care Medicine and Pain Therapy, University Hospital Giessen, UKGM, Justus‐Liebig University Giessen, Giessen, Germany; 14 Institute of Biometry and Clinical Epidemiology, Charité – Universitätsmedizin Berlin, Corporate Member of Freie Universität Berlin and Humboldt-Universität zu Berlin, Berlin, Germany; 15 Institute of Medical Informatics, Charité – Universitätsmedizin Berlin, Corporate Member of Freie Universität Berlin and Humboldt-Universität zu Berlin, Berlin, Germany; 16 Department of Neurology and Center for Translational Neuro- and Behavioral Sciences, University Hospital Essen, Essen, Germany

**Keywords:** Critical care, Delirium, Delivery of health care, Europe, Intensive care units

## Abstract

**Background:**

The objective of this study was to analyze the impact of a structured educational intervention on the implementation of guideline-recommended pain, agitation, and delirium (PAD) assessment.

**Methods:**

This was a prospective, multinational, interventional before-after trial conducted at 12 intensive care units from 10 centers in Germany, Austria, Switzerland, and the UK. Intensive care units underwent a 6-week structured educational program, comprising online lectures, instructional videos, educational handouts, and bedside teaching. Patient-level PAD assessment data were collected in three 1-day point-prevalence assessments before (T1), 6 weeks after (T2), and 1 year after (T3) the educational program.

**Results:**

A total of 430 patients were included. The rate of patients who received all three PAD assessments changed from 55% (107/195) at T1 to 53% (68/129) at T2, but increased to 73% (77/106) at T3 (*p* = 0.003). The delirium screening rate increased from 64% (124/195) at T1 to 65% (84/129) at T2 and 77% (82/106) at T3 (*p* = 0.041). The pain assessment rate increased from 87% (170/195) at T1 to 92% (119/129) at T2 and 98% (104/106) at T3 (*p* = 0.005). The rate of sedation assessment showed no signficiant change. The proportion of patients who received nonpharmacological delirium prevention measures increased from 58% (114/195) at T1 to 80% (103/129) at T2 and 91% (96/106) at T3 (*p* < 0.001). Multivariable regression revealed that at T3, patients were more likely to receive a delirium assessment (odds ratio [OR] 2.138, 95% confidence interval [CI] 1.206–3.790; *p* = 0.009), sedation assessment (OR 4.131, 95% CI 1.372–12.438; *p* = 0.012), or all three PAD assessments (OR 2.295, 95% CI 1.349–3.903; *p* = 0.002) compared with T1.

**Conclusions:**

In routine care, many patients were not assessed for PAD. Assessment rates increased significantly 1 year after the intervention.

*Clinical trial registration* ClinicalTrials.gov: NCT03553719.

**Supplementary Information:**

The online version contains supplementary material available at 10.1007/s12028-023-01837-8.

## Background

Pain, agitation/sedation, and delirium (PAD) are common among critically ill patients. Previous studies have shown that pain is experienced by about half of medical and surgical intensive care unit (ICU) patients at rest [1, 2], severe or dangerous agitation is present in about half of ICU patients [3], 27% of ICU patients have been found to be deeply sedated [4], and up to 82% of ICU patients undergoing mechanical ventilation are affected by delirium [5, 6]. PAD are detrimental to patient outcomes. High-quality pain management reduces the duration of mechanical ventilation and the nosocomial infection rate [7], and early deep sedation is an independent predictor for delayed extubation and 6-month mortality [8]. Delirium is associated with a longer duration of mechanical ventilation [6], longer hospitalization [5, 6], increased mortality [9, 10], and higher rates of long-term cognitive impairment [11]. Hence, PAD management is an integral part of intensive care [12–14].

Guidelines recommend that PAD should be assessed every 8 h using validated screening tools [12–14]. Self-assessment is useful among patients able to report pain (e.g., the Numeric Rating Scale [NRS]) [15], alternatively observational tools such as the Behavioral Pain Scale (BPS) [16, 17] or the Critical-Care Pain Observation Tool for those unable to [12–14, 18]. Guidelines consider the Confusion Assessment Method for the Intensive Care Unit (CAM-ICU) [19] and the Intensive Care Delirium Screening Checklist (ICDSC) [20] as the most suitable delirium screening instruments and recommend the Richmond Agitation Sedation Scale (RASS) [21] or the Sedation Agitation Scale [22] for sedation assessment [12–14].

Despite the relevance of evidence-based PAD assessment, previous studies from routine care have indicated poor implementation rates in various settings. In a multinational survey in 2019/20 among a convenience sample of 1474 intensivists, 95.4% of respondents reported assessing delirium daily, but two thirds only assessed patients they considered to be at risk [23]. Although 85.4% of participants responded to assess sedation levels and 86.7% responded to assess pain in patients able to communicate, only two thirds assessed pain in those unable to communicate [23]. In a 2014 survey among 101 European ICUs, 49%, 30%, and 79% of centers reported the 8-hourly assessments of pain, agitation or delirium, respectively, but under half of patients in this study actually received PAD monitoring [24]. These results mirror surveys from Poland in 2016 [25], Belgium in 2011 [26], Australia/New Zealand in 2006/7 [27], Canada in 2002 [28], and Germany in 2002 [29].

Structured face-to-face training and e-learning have been employed as effective strategies to increase staff knowledge and improve the frequency and quality of routine PAD assessment in previous studies [30–37]. A prospective cohort study on three German ICUs of one university hospital showed an improvement in PAD screening rates more than 1 year following the implementation of formal and bedside teaching as well as the provision of educational resources and delirium support teams [38].

We are not aware of recent data on the real-world, guideline-adherent PAD assessment rates in European ICUs. In this prospective before-after trial among patients of 12 European ICUs, we first investigated whether PAD assessment rates change 6 weeks and 1 year following a structured educational program. Second, we explored which patient-specific characteristics might determine whether a patient was assessed for pain, sedation, or delirium.

## Methods

### Study Design

**Fig. 1:**
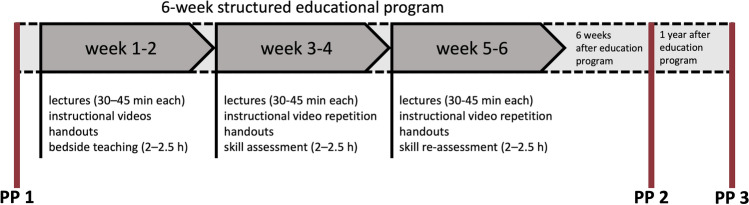
Concept of the structured educational program and data collection at assessment points (red lines). *PP* point prevalence

In this prospective, multinational, interventional before-after trial, data on the assessment of delirium, analgesia, and sedation for inpatients in participating adult ICUs were collected at three 1-day (i.e., 24-h) point-prevalence assessments. Immediately after the first 1-day assessment from December 2018 to January 2019, study sites underwent a structured 6-week educational program, aimed at improving the frequency and quality of PAD assessments. The educational program was based on a previously published extensive training algorithm [38]. Data for two additional 1-day point prevalences were collected 6 weeks (May–June 2019) and 1 year (March–May 2020) after the conclusion of the educational program (Fig. 1). The study adhered to the ethical standards of the Declaration of Helsinki and its later amendments and its protocol was registered at ClinicalTrials.gov on June 12, 2018 (Identifier: NCT03553719). Ethical approval at the coordinating study site was granted by the Institutional Review Board (IRB) of Charité–Universitätsmedizin Berlin (EA2/022/18) on March 23, 2018. Participating centers obtained their IRB approval before study commencement as required by local regulations. Written consent or a waiver was subject to the decision of the local IRBs.

### Trial ICUs and Participants

The trial was hosted by Charité–Universitätsmedizin Berlin and conducted at 12 European adult ICUs from 10 centers, located in Germany, the UK, Austria, and Switzerland (Table S1). Centers were recruited from members of the Working Group Postoperative Delirium and Cognitive Dysfunction of the European Society of Intensive Care Medicine and its NEXT Committee.

Patients qualified for inclusion if they were treated in a participating ICU on a day of a point-prevalence assessment and were ≥ 18 years of age. Patients were excluded for blindness, deafness, or an insurmountable language barrier.

### Structured Educational Program

We applied a structured educational algorithm pertaining to PAD based on a previously described training algorithm [38]. In short, the educational program consisted of three 2-week cycles and was based on the train-the-trainer concept. It combined theoretical lectures, handouts with key points, instructional videos, and bedside teaching. On the first 4 days of each cycle, trained PAD experts from Charité (MR and BW) provided participants of all study centers with 30–45-min webinars on the assessment of sedation and pain, the minimization of sedation, the pathophysiology, symptomatology, prevention, and detection of delirium, and provided a demonstration of the CAM-ICU. These webinars were recorded and provided to participants for later review and dissemination. All informational content was distributed to local study coordinators to adapt into their units’ practice, providing the core messages and structure remained unchanged. On the fifth day of each cycle, theoretical teaching was followed by 2–2.5-h face-to-face bedside teaching from PAD experts at each participating center, with immediate feedback and debriefing for participants. PAD experts were consultants with expertise in pain, sedation and delirium, and they were part of the study team. Bedside teaching also addressed a potential lack of documentation of assessments. At three centers where the local PAD expert was not available, bedside teaching was provided by a PAD expert from Charité (MR or BW). In the second week of each cycle, ICU staff were expected to apply the learning content in routine care. Our overarching goal was to train local “PAD leaders.” Local project leaders were tasked with recruting interested staff members. Participation was voluntary and open to all ICU staff members (doctors, nurses, and other health care professionals), but most participants were ICU nurses. Every center received its individual training to keep group sizes small and lower the barriers for questions during the training. Training of centers was performed sequentially and in close temporal proximity (Fig. 1).

### Outcomes

The primary outcome was the percentage of patients screened for delirium at least once on the day of the point-prevalence assessment (delirium screening rate). Secondary outcomes comprised percentage of patients who received at least one pain or sedation assessment on the day of the point-prevalence assessment (pain and sedation assessment rates), delirium, pain, and sedation screening tools used, respective pain and sedation scores, delirium prevalence in routine delirium screening, and nonpharmacological measures to prevent or treat delirium (i.e., reorientation method, early mobilization, and sensory shielding) [39–41].

### Data Collection

Patient characteristics, treatment data, and PAD management were recorded for each patient in an electronic case report form (LimeSurvey, Hamburg, Germany). Data on PAD assessment were taken from the patients’ health care records to obtain information on the current ICU practice. The assessors were therefore ICU physicians and nurses as per local standard. Data were stored on a secure server on the premises of the coordinating study site and handled according to European Union General Data Protection Regulations. Except for local study coordinators, ICU staff was unaware of the assessment days. Local study coordinators were also prompted to pick days that were not particularly busy or had usual staffing.

### Data Analysis

In previously published work [24], 27% of patients received routine delirium screening prior to an educational program, and in a study implementing an educational program similar to our proposed intervention, the routine delirium screening rate increased by about 15% after a month of training [42]. According to our a priori sample size calculation, we could detect an increase in screening rate from 27% before the educational program to 42% following the educational program if each participating ICU enrolled 14 patients on average (170 patients total) per assessment point, with a power of at least 0.8 and a two-tailed significance level of 0.05 (Fisher’s exact test; nQuery Advisor 7.0, Statsols, Cork, Ireland). Although sample size planning was done conservatively on the basis of a simple two-group test, we planned to account for the clustered data structure in the analysis.

Descriptive statistics of the study population are either presented as medians with limits of the interquartile range (IQR) or absolute (*n*) with relative frequencies (%). Patient characteristics and PAD assessment points were compared using Pearson’s *χ*^2^ tests for categorical variables and Kruskal–Wallis test for continuous variables. Adjustments were not made for multiple testing. Multivariable mixed-effect logistic regression analyses were used to assess the association between the assessment points and the likelihood for a patient to receive a delirium, sedation, or pain assessment, all PAD assessments, or nonpharmacological measures to prevent or treat delirium, respectively. The regression models included sex, age, extracorporeal membrane oxygenation provision (yes/no), and mechanical ventilation (yes/no) as fixed effects. To account for the clustered nature of the data, a random effect for the treating country was added (Germany, Austria, Switzerland, or the UK). Statistical analyses were performed using Stata 17SE (StataCorp LP, College Station, TX).

## Results

### Study Population

A total of 430 patients from 12 ICUs from 10 centers were included, with 195 patients at the first (December 2018–January 2019), 129 patients from 8 centers at the second (May–June 2019), and 106 patients from 6 centers at the third (March–May 2020) 24-h assessment point. Recruitment by center and assessment point is shown in Table S1.

At the first, second, and third assessment points 76%, 66%, and 72% of patients, respectively, were mechanically ventilated. The median Simplified Acute Physiology Score II scores at admission were 37 (IQR 27–53), 41.5 (IQR 31.5–49), and 40 (IQR 28–51). There were no significant differences between patients of the three assessment points with respect to age, sex, height, weight, admitting diagnosis, current length of ICU stay, organ support received, mechanical ventilation, extracorporeal membrane oxygenation provision, and severity of illness (Table 1).

**Table 1:** Characteristics of the study population

Variable	Assessment point			*p* value
	1 (*n* = 195)	2 (*n* = 129)	3 (*n* = 106)	
Age (years)	63 (52–73)	59 (52–73)	64 (52–74)	0.552^a^
Female sex	75 (38%)	41 (32%)	47 (44%)	0.173^b^
BMI (kg/m^2^)	26.4 (23.2–30.7)	26.2 (23.9–29.7)	25.7 (23.1–29.4)	0.513^a^
Primary ICU admission diagnosis				
Respiratory	43 (22%)	26 (20%)	21 (20%)	0.107^b^
Septic/infectious	20 (10%)	12 (9%)	12 (11%)	
Gastrointestinal	28 (14%)	9 (7%)	15 (14%)	
Cardiovascular	45 (23%)	45 (35%)	23 (22%)	
Traumatic	24 (12%)	6 (5%)	8 (8%)	
Neurologic	17 (9%)	18 (14%)	17 (16%)	
Metabolic or endocrine	2 (1%)	4 (3%)	4 (4%)	
Oncologic	9 (5%)	3 (2%)	3 (3%)	
Others^c^	7 (4%)	6 (5%)	3 (3%)	
ICU length of stay at day of assessment (d)	7 (3–17)	7 (2–15)	7 (3–15)	0.819^a^
Received any organ support	156 (80%)	98 (76%)	83 (78%)	0.65^b^
Mechanical ventilation	147 (76%)	89 (66%)	78 (72%)	0.321^b^
ECMO	19 (10%)	20 (16%)	7 (7%)	0.083^b^
RASS on assessment day	0 (− 2–0)	− 1 (− 3.5–0)	− 1 (− 3–0)	0.087^a^
SAPS II at admission	37 (27–53)	41.5 (31.5–49)	40 (28–51)	0.709^a^
SOFA at admission	7 (4–10)	8 (5–12)	7 (4–10)	0.247^a^

*n* (% of patients at respective assessment point) or median (25th percentile to 75th percentile). Ten centers participated at assessment point 1, eight centers participated at assessment point 2, and six centers participated at assessment point 3

*BMI* body mass index, *ECMO* extracorporeal membrane oxygenation, *ICU* intensive care unit, *RASS* Richmond Agitation Sedation Scale, *SAPS II* Simplified Acute Physiology Score II, *SOFA* Sequential Organ Failure Assessment Score

^a^Kruskal-Wallis test

^b^Pearson’s *χ*^2^ test

^c^Others include e.g. acute kidney injury, laryngectomy, Cesarean section, autosomal dominant polycystic kidney disease, renal transplant, neck dissection, or sickle cell crisis

### Delirium, Sedation, and Pain Assessments

**Fig. 2:**
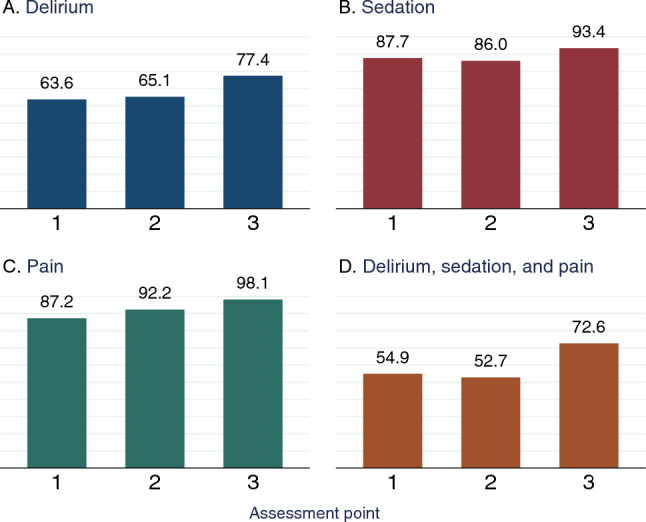
Percentage of patients who received a **a** delirium screening, **b** sedation assessment, **c** pain assessment, or **d** complete PAD assessment (delirium, sedation, and pain), by assessment point. In percentages. Assessment point 1 comprised *n* = 195 patients. Assessment point 2 comprised *n* = 129 patients. Assessment point 3 comprised *n* = 106 patients. Pearson’s *χ*^2^ test for differences between assessment points for delirium screening (*p* = 0.041), sedation assessment (*p* = 0.182), pain assessment (*p* = 0.005), and complete PAD assessment (*p* = 0.003). *PAD* pain, agitation, and delirium

Delirium screening was performed in 64% (124/195) of patients at least once daily at baseline. The CAM-ICU was used in 56% (109/195) of patients, the nursing delirium screening scale in 4% (7/195), and the ICDSC in 5% (10/195) of patients. Two patients received both CAM-ICU and ICDSC assessments. After completion of the educational program, the delirium screening rate increased to 65% (84/129) at 6 weeks, and 77% (82/106) at 1 year (*p* = 0.041). Among those screened, 26% (32/124), 23% (19/84), and 13% (11/82) screened positive for delirium at assessment points 1, 2, and 3, respectively. Screening for delirium at assessment points 2 and 3 was conducted solely using the CAM-ICU, as the two centers using the ICDSC and nursing delirium screening scale at assessment point 1 did not recruit patients at later assessment points (Table 2, Fig. 2a, Fig. S1a).

**Table 2:** Characteristics of delirium, pain, and sedation assessments

Variable	Assessment point			*p* value^a^
	1 (*n* = 195)	2 (*n* = 129)	3 (*n* = 106)	
Delirium screening with validated tool on assessment day	124 (64%)	84 (65%)	82 (77%)	**0.041**
CAM-ICU used	109 (56%)^b^	84 (65%)	82 (77%)	**0.001**
Nu-DESC used	7 (4%)^c^	0 (0%)	0 (0%)	–
ICDSC used	10 (5%)^b,d^	0 (0%)	0 (0%)	–
Positive delirium screening, *n* (% of those screened)	32 (26%)	19 (23%)	11 (13%)	0.099
Sedation depth assessed with validated tool on assessment day	171 (88%)	111 (86%)	99 (93%)	0.182
RASS used	169 (87%)	111 (86%)	78 (74%)	**0.009**
SAS used	0 (0%)	0 (0%)	0 (0%)	–
Other sedation scale used	2 (1%)	0 (0%)	21 (20%)^e^	**< 0.001**
Pain assessed with validated tool on assessment day	170 (87%)	119 (92%)	104 (98%)	**0.005**
VAS used	12 (6%)	5 (4%)	4 (4%)	0.538
NRS used	89 (46%)	50 (39%)	54 (51%)	0.167
BPS used	47 (24%)	31 (24%)	42 (40%)	**0.008**
BPS-NI used	5 (3%)	5 (4%)	1 (1%)	0.367
CPOT used	16 (8%)^f^	18 (14%)^f^	0 (0%)	–
Delirium, sedation, and pain assessed on assessment day	107 (55%)	68 (53%)	77 (73%)	**0.003**
Nonpharmacological measures to prevent or treat delirium	114 (58%)	103 (80%)	96 (91%)	**< 0.001**
Sensory shielding	47 (24%)	41 (32%)	33 (31%)	0.236
Reorientation (e.g., clock or whiteboard)	87 (45%)	68 (53%)	71 (67%)	**0.001**
Early mobilization	83 (43%)	59 (46%)	61 (58%)	**0.042**

Bold values represent significant *p* values with *p* < 0.05

*n* (% of patients at assessment point) if not indicated otherwise. Ten centers participated at assessment point 1, eight centers participated at assessment point 2, and six centers participated at assessment point 3

*CAM-ICU* Confusion Assessment Method for the Intensive Care Unit, *BPS* Behavioral Pain Scale, *BPS-NI* Behavioral Pain Scale–Nonintubated, *CPOT* Critical Care Pain Observation Tool, *ICDSC* Intensive Care Delirium Screening Checklist, *NRS* Numeric Rating Scale, *Nu-DESC* Nursing Delirium Screening Scale, *SAS* Sedation Agitation Scale, *RASS* Richmond Agitation Sedation Scale, *VAS* Visual Analogue Scale

^a^Pearson’s *χ*^2^ test if not indicated otherwise

^b^*n* = 2 patients received both, a CAM-ICU assessment and an ICDSC assessment. In both patients, assessments were negative

^c^The Nu-DESC was applied by one German center that did not recruit patients at assessment points 2 and 3

^d^The ICDSC was applied by one German center that did not recruit patients at assessment points 2 and 3

^e^The Alert, Verbal, Pain, Unresponsive (AVPU) scale was applied in 21 patients in one UK center at assessment point 3

^f^The CPOT was applied by one UK center that did not recruit patients at assessment point 3

At baseline, 58% (114/195) of patients received nonpharmacological measures to prevent or treat delirium. After the educational program, 80% (103/129) received nonpharmacological measures at assessment point 2, and 91% (96/106) at assessment point 3 (*p* < 0.001). The most common measure was reorientation (e.g., clock or whiteboard), which was used in 45% (87/195), 53% (68/129), and 67% (71/106) of patients at assessment points 1, 2, and 3, respectively (*p* = 0.001). The second most common nonpharmacological measure was early mobilization, which was provided to 43% (83/195), 46% (59/129), and 58% (61/106) of patients at assessment points 1, 2, and 3, respectively (*p* = 0.042).

Sedation depth was assessed in 88% (171/195) of patients at least once per day at baseline. After the educational program, 86% (111/129) received sedation depth assessment at assessment point 2, and 93% (99/106) at assessment point 3 (*p* = 0.182). RASS was used in 87% (169/195), 86% (111/129), and 74% (78/106) of patients (*p* = 0.009). The Sedation Agitation Scale was not used in any ICUs. Other sedation scales included the alert, verbal, pain, unresponsive scale, which was used in 20% (21/106) of patients, all of which were treated in one UK center at assessment point 3 (Table 2, Fig. 2b, Fig. S1c).

Pain was evaluated in 87% (170/195) of patients at least once per day at baseline. After completion of the educational program, the rate increased to 92% (119/129) and 98% (104/106) at assessment points 2 and 3, respectively (*p* = 0.005). The NRS was applied most frequently (46%, 39%, and 51% of patients at assessment points 1, 2, and 3, respectively), followed by the BPS (24%, 24%, and 40% of patients). The critical-care pain observation tool was only used by one UK center, which recruited patients at assessment points 1 and 2, but not 3 (Table 2, Fig. 2c, Fig. S1b).

At assessment point 1, 55% (107/195) of patients received all PAD assessments (pain, sedation, and delirium) at least once per day. Six weeks after completion of the educational program, 53% (68/129) of patients received all PAD assessments, which increased to 73% (77/106) at 1 year (*p* = 0.003; Table 2, Fig. 2d, Fig. S1d, Fig. S2). As shown in Fig. S1, centers started at different assessment rates for all PAD assessments, and while some centers improved, others stayed the same or worsened.

### Determinants of Delirium, Pain, and Sedation Assessment

Multivariable mixed-effects logistic regression revealed that patients were more than twice as likely to receive delirium screening 1 year after the educational program (odds ratio [OR] 2.138, 95% confidence interval [CI] 1.206–3.790; *p* = 0.009) compared with before. Mechanical ventilation (OR 0.266, 95% CI 0.147–0.481; *p* < 0.001) or male sex (OR 0.635, 95% CI 0.404–0.997; *p* = 0.049) lowered the likelihood to receive delirium screening in our adjusted model. Compared with before, patients were more than twice as likely to receive nonpharmacological measures for delirium treatment or prevention 6 weeks after the educational program (OR 2.751, 95% CI 1.592–4.753; *p* < 0.001), and almost 10 times as likely 1 year after the educational program (OR 9.943, 95% CI 4.436–22.282; *p* < 0.001). The odds for sedation assessment were four times higher 1 year after the educational program (OR 4.131, 95% CI 1.372–12.438; *p* = 0.012) compared with before. Mechanical ventilation further increased the odds for sedation assessment (OR 5.684, 95% CI 2.765–11.683; *p* < 0.001). Almost all patients (98%) received pain assessment 1 year after the educational program. Finally, patients were more than two times more likely to receive all PAD assessments after 1 year (OR 2.295, 95% CI 1.349–3.903; *p* = 0.002) compared with before (Table 3).

**Table 3:** Multivariable mixed-effects logistic regression on the assessment of delirium, sedation, and/or pain, and the use of nonpharmacological measures to prevent or treat delirium

Variable	Delirium assessed with validated screening tool		Sedation assessed with validated tool		Pain assessed with validated tool		Delirium, sedation, and pain assessed		Nonpharmacological measures to prevent or treat delirium used^a^	
	Odds ratio (95% CI)	*p* value	Odds ratio (95% CI)	*p* value	Odds ratio (95% CI)	*p* value	Odds ratio (95% CI)	*p* value	Odds ratio (95% CI)	*p* value
Male sex	0.635 (0.404; 0.997)	**0.049**	1.183 (0.576; 2.429)	0.647	0.560 (0.246; 1.275)	0.167	0.780 (0.514; 1.184)	0.244	0.809 (0.494; 1.326)	0.401
Age (years)	0.998 (0.984; 1.011)	0.723	1.001 (0.979; 1.024)	0.902	0.985 (0.959; 1.011)	0.258	1.001 (0.988; 1.013)	0.935	0.994 (0.979; 1.009)	0.447
ECMO, yes	0.660 (0.337; 1.290)	0.224	6.008 (0.753; 47.955)	0.091	0.574 (0.149; 2.210)	0.42	0.750 (0.384; 1.465)	0.4	1.158 (0.523; 2.567)	0.717
Mechanical ventilation, yes	0.266 (0.147; 0.481)	**< 0.001**	5.684 (2.765; 11.683)	**< 0.001**	1.069 (0.391; 2.923)	0.896	0.828 (0.503; 1.361)	0.456	0.869 (0.478; 1.582)	0.647
Assessment point 1 (reference)	1	–	1	–	1	–	1	–	1	–
Assessment point 2	0.990 (0.603; 1.626)	0.969	0.886 (0.413; 1.903)	0.757	1.393 (0.593; 3.273)	0.447	0.901 (0.565; 1.437)	0.662	2.751 (1.592; 4.753)	**< 0.001**
Assessment point 3	2.138 (1.206; 3.790)	**0.009**	4.131 (1.372; 12.438)	**0.012**	NA	NA^b^	2.295 (1.349; 3.903)	**0.002**	9.943 (4.436; 22.282)	**< 0.001**
Constant	12.464 (3.571; 43.501)	**< 0.001**	4.656 (0.366; 59.312)	0.236	128.239 (7.040; 2,336.128)	**0.001**	2.127 (0.662; 6.834)	0.205	2.814 (0.596; 13.284)	0.191

To account for the clustered data, a random effect for the treating country was included in the regression models

Bold values represent significant *p* values with *p* < 0.05

*CI* confidence interval, *ECMO* extracorporeal membrane oxygenation, *NA* not available

^a^Nonpharmacological measures to prevent or treat delirium comprised reorientation, early mobilization, and/or sensory shielding

^b^At assessment point 3, 104/106 (98%) of patients received a pain assessment. Hence, no odds ratio could be estimated

In sensitivity analyses, we first excluded two centers that only recruited patients at assessment point 1 from the analysis. This reduced the screening rate of delirium, pain, sedation, and all PAD assessments for assessment period 1, and resulted in increased effects observed in the multivariable regression (Tables S2 and S3). Second, we excluded four centers that did not recruit at all assessment points from the analysis. This resulted in increased odds of a sedation and complete PAD assessment at assessment point 3, but the significant association of assessment point 3 with the odds of a delirium screening disappeared. Yet, assessment point 2 was newly associated with higher odds of a pain and complete PAD assessment (Tables S4 and S5).

## Discussion

In this prospective before-after trial, we examined PAD assessment rates among patients of 12 European ICUs before and after the implementation of a structured educational intervention. About two thirds of patients were screened for delirium at baseline (64%) and 6 weeks after the educational program (65%), which increased to 77% after 1 year. Further, before the intervention, 55% of patients received a complete PAD assessment, which increased to almost three quarters (73%) 1 year after the intervention. Multivariable regression revealed that patients were more than twice as likely to receive a complete PAD assessment 1 year after the educational program.

PAD assessment rates in routine ICU care have been analyzed in previous studies. A study on ABCDEF (Awakening and Breathing Coordination, Delirium monitoring/management, Early exercise/mobility, Family engagement) bundle implementation among critically ill patients with Covid-19 found pain, sedation, and delirium assessment rates of 45%, 52%, and 35%, respectively [43]. These rates are much lower compared to the third assessment point in our study, which fell in the Covid-19 pandemic. However, contrary to our study, participating ICUs did not undergo a structured training, and they only included patients with Covid-19. Because of isolation and preventive measures, PAD assessment and mobilization may have been more difficult for patients with Covid-19 than for patients without Covid-19. In a recent international survey, 85.4% of the 1474 intensivists reported using sedation scales [23]. Just like in our study, the RASS was the commonest. Almost all intensivists (95.4%) reported assessing patients for delirium at least once per day [23], which is contrary to our measured findings and may be indicative of a discrepancy between surveys among ICU staff and real-world point-prevalence studies. In another point-prevalence estimation among a convenience sample of European ICU patients in 2011, more than half (57%) of the 868 patients were not assessed for pain or sedation depth on the study day [24], and almost three quarters (73.1%) were not screened for delirium with a validated instrument. Those rates are below the baseline assessment rates found in our study, where almost nine of ten patients received a pain and/or sedation assessment and 64% were screened for delirium. Another survey among 165 Polish ICUs from 2016 showed that only 10.9% of ICUs monitored delirium and 46.1% reported using sedation scales [25]. The sedation scale most used was the Ramsey scale, and delirium was most commonly identified using the International Classification of Diseases, 10th revision, whereas in our study, the RASS and CAM-ICU were most used [25]. In another survey among 214 ICUs in the UK in 2013/14, 57% of ICUs reported having a sedation protocol, 69.7% reported daily delirium screening, and 93.4% reported routine application of sedation scales [44], which appears consistent with our findings.

We found at 1 year after the educational program, PAD assessment rates significantly improved compared with baseline, with no effect seen at 6 weeks. These findings are consistent with a study conducted among three ICUs of one German hospital, which were subject to similar PAD training consisting of lectures, educational material, and tailored bedside teaching [38]. Similar to our study, their reported frequency of daily PAD monitoring significantly increased more than a year after the training [38]. In contrast to our study, they observed already improvements about 4 weeks after the training. However, their training was twice as long, they had a permanent on-site support team, and they trained all ICU staff instead of using a train-the-trainer concept. The observed delay in improvement in our study could possibly be explained by the train-the-trainer concept and the organizational nature of an ICU, as more than 6 weeks are likely required for the trainer to have sufficient face-to-face time with other staff members to disseminate and implement the content. This explanation is supported by reports on the challenges of changing routine actions and putting new evidence into practice [45]. Our results appear even more noteworthy considering that the third assessment point coincided with the Covid-19 pandemic, which put pressure on already constrained ICU resources. In the absence of the Covid-19 pandemic, we might have observed a stronger increase in PAD assessment rates. Alternatively, the Covid-19 pandemic may have prompted hospitals to focus more on intensive care practice.

PAD assessment is the important first step of adequate pain control, sedation management and delirium prevention, which is known to improve patient outcomes. Systematic pain and sedation assessments three times per day and after painful procedures have been shown to decrease the incidence of severe pain, the duration of mechanical ventilation, and nosocomial infections on a surgical ICU [7]. In a single-center study, an educational intervention using lectures and posters was used to implement an updated protocol mandating documented sedation assessment every 4 h, documented delirium assessment twice daily, and protocolized sedative dose reductions for patients with RASS − 2 or − 3 [46]. After implementation, patients had increased PAD assessment rates, reduced excess sedation, shorter mechanical ventilation, reduced ICU and hospital lengths of stay, and a lower risk of developing delirium [46]. High-quality PAD assessment and management should be part of a broader bundled quality improvement approach such as the ABCDE bundle. Rigorous application of the ABCDE bundle was shown to be associated with lower odds of delirium, more time breathing without mechanical assistance, and a greater likelihood of mobilization [47]. Interestingly, with increasing delirium screening rates, we observed a decline in positive delirium screenings from 26% at assessment point 1 to 13% at assessment point 3. This may indicate that 1 year after the training, a broad cohort of patients received a validated screening, and not only those patients appearing conspicuous of having delirium. Additionally, the higher rate of patients who received nonpharmacological measures to prevent or treat delirium 1 year after the intervention may have reduced the delirium incidence.

The strengths of this study include the international multicenter study design in a distinct geographical region. This enabled us to capture a range of PAD management practices in Europe. Patients were recruited prospectively, and patients treated on a particular day and ICU were enrolled. This should reduce the selection bias inherent to previous studies of routine PAD assessment that used cross-sectional surveys and convenience sampling [24, 27–29]. However, our findings come with limitations. Our before-after trial design does not allow for causal inferences on the effects of the educational program as external effects between time points one and three cannot be excluded. That is, we may have detected a general trend of improved PAD assessments or improved documentation over time, independent from our educational program. Further, we considered PAD assessments documented in the medical records. However, patients may have been assessed without documentation, although documentation is part of a complete formal assessment. Also, we did not collect longitudinal data on PAD management in patients throughout their ICU stay, but rather captured three cross-sectional time points. The train-the-trainer concept may have impeded penetration of the educational program, especially because we did not track how many training participants worked in the respective ICU at the 1-year follow-up. In addition, we observed a discrepancy between ICU bed capacity and patient enrollment in many centers, but it is uncertain if these ICUs were running below capacity on the assessment days or if some patients were not enrolled. Furthermore, we determined the rate of patients receiving PAD assessment, but an analysis of the quality or accuracy of these assessments and changes in PAD therapy, outcomes, or adverse events was beyond our study’s scope. Two centers ceased recruiting for the second assessment point and another two centers ceased recruiting at the third assessment point due to the onset of the Covid-19 pandemic in March 2020. In response, we conducted sensitivity analyses where we excluded the center dropouts to exclude the attrition bias. Finally, baseline delirium assessment rates were higher than we had anticipated based on previous literature. This may be due to improvements in delirium screening over time or may suggest that included centers already had above-average delirium screening rates.

## Conclusions

In routine care, only about half of included ICU patients received a complete PAD assessment. Six weeks after a PAD educational program, we observed no significant improvement of PAD assessment rates, but a significant improvement 1 year after the educational program. The instruments most frequently used were the CAM-ICU for delirium, the RASS for sedation, and either the NRS or BPS for pain. Notably, at 6 weeks and at 1 year after the educational program, significantly more patients received nonpharmacological measures to prevent or treat delirium. Future randomized cohort studies should analyze the time lag of educational programs to cause behavioral changes and confirm that educational programs effectively improve PAD assessment rates.

### Supplementary Information

Below is the link to the electronic supplementary material.

Supplementary file1 (DOCX 273 KB)
