# Supplementary material for: Enhancing European Management of Analgesia, Sedation, and Delirium: A Multinational, Prospective, Interventional Before-After Trial
Source: Neurocrit Care. 2023 Sep 11;40(3):898–908. doi: 10.1007/s12028-023-01837-8 (PMC11147880; doi:10.1007/s12028-023-01837-8)
Supplement: Supplementary file 1 — Supplementary file1 (DOCX 273 KB) [file 12028_2023_1837_MOESM1_ESM.docx]

**Supplementary material: 5 tables, 2 figures**

**Table S1.** Patient recruitment by center.

| **Center** | **Number of ICUs** | **ICU type** | **ICU beds** | **University hospital** | **Country** | **Assessment point, n** | | |
| --- | --- | --- | --- | --- | --- | --- | --- | --- |
|  |  |  |  |  |  | **1** | **2** | **3** |
| 1 | 3 | surgical | 51 | Yes | Germany | 36 | 22 | 30 |
| 2 | 1 | mixed | 36 | No | Switzerland | 16 | 13 | 16 |
| 3 | 1 | mixed | 40 | Yes | UK | 34 | 22 | 34 |
| 4 | 1 | medical | 8 | Yes | Austria | 4 | 7 | 6 |
| 5 | 1 | mixed | 46 | Yes | UK | 46 | 37 | 0 |
| 6 | 1 | surgical | 16 | Yes | Germany | 13 | 9 | 0 |
| 7 | 1 | surgical | 24 | Yes | Germany | 14 | 0 | 0 |
| 8 | 1 | surgical | 13 | Yes | Germany | 9 | 0 | 0 |
| 9 | 1 | neurological | 10 | Yes | Germany | 10 | 8 | 10 |
| 10 | 1 | mixed | 19 | Yes | UK | 13 | 11 | 10 |
| **Total** |  |  |  |  |  | **195** | **129** | **106** |

**Table S2.** Characteristics of delirium, pain, and sedation assessments, excluding data from centers 7 and 8 that only recruited patients at assessment point 1.

| **Variable** | **Assessment point** | | | ***p* ^a^** |
| --- | --- | --- | --- | --- |
|  | **1**  **(n = 172)** | **2**  **(n = 129)** | **3**  **(n = 106)** |  |
| Delirium screening with validated tool on assessment day | 105 (61 %) | 84 (65 %) | 82 (77 %) | **0.018** |
| CAM-ICU used | 105 (61 %) | 84 (65 %) | 82 (77 %) | **0.018** |
| Nu-DESC used | 0 (0 %) | 0 (0 %) | 0 (0 %) | - |
| ICDSC used | 0 (0 %) | 0 (0 %) | 0 (0 %) | - |
| Positive delirium screening,  n (% of those screened) | 20 (19 %) | 19 (23 %) | 11 (13 %) | 0.099 |
| Sedation depth assessed with validated tool on assessment day | 148 (86 %) | 111 (86 %) | 99 (93 %) | 0.135 |
| RASS used | 146 (85 %) | 111 (86 %) | 78 (74 %) | **0.023** |
| SAS used | 0 (0 %) | 0 (0 %) | 0 (0 %) | - |
| Other sedation scale used | 2 (1 %) | 0 (0 %) | 21 (20 %) ^b^ | **< 0.001** |
| Pain assessed with validated tool on assessment day | 148 (86 %) | 119 (92 %) | 104 (98 %) | **0.002** |
| VAS used | 12 (7 %) | 5 (4 %) | 4 (4 %) | 0.366 |
| NRS used | 80 (47 %) | 50 (39 %) | 54 (51 %) | 0.158 |
| BPS used | 34 (20 %) | 31 (24 %) | 42 (40 %) | **0.001** |
| BPS-NI used | 5 (3 %) | 5 (4 %) | 1 (1 %) | 0.501 |
| CPOT used | 16 (9 %) ^c^ | 18 (14 %) ^c^ | 0 (0 %) | - |
| Delirium, sedation, and pain assessed on assessment day | 89 (52 %) | 68 (53 %) | 77 (73 %) | **0.001** |
| Non-pharmacological measures to prevent or treat delirium | 101 (59 %) | 103 (80 %) | 96 (91 %) | **< 0.001** |
| Sensory shielding | 43 (25 %) | 41 (32 %) | 33 (31 %) | 0.358 |
| Reorientation (e.g. clock or whiteboard) | 75 (44 %) | 68 (54 %) | 71 (67 %) | **0.001** |
| Early mobilization | 71 (41 %) | 59 (46 %) | 61 (58 %) | **0.029** |

n (% of patients at assessment point) if not indicated otherwise. 8 centers participated at assessment point 2, and 6 centers participated at assessment point 3. ^a^ Pearson’s χ² test if not indicated otherwise. ^b^ The AVPU (alert, verbal, pain, unresponsive) scale was applied in 21 patients in one UK center at assessment point 3. ^c^ The CPOT was applied by one UK center which did not recruit patients at assessment point 3. CAM-ICU, Confusion Assessment Method for the Intensive Care Unit; BPS, Behavioral Pain Scale; BPS-NI, Behavioral Pain Scale – Non-Intubated; CPOT, Critical Care Pain Observation Tool; ICDSC, Intensive Care Delirium Screening Checklist; NRS, Numeric Rating Scale; Nu-DESC Nursing Delirium Screening Scale; SAS, Riker Sedation Agitation Scale; RASS, Richmond Agitation Sedation Scale; VAS, Visual Analogue Scale.

**Table S3.** Multivariable mixed-effects logistic regression on the assessment of delirium, sedation, and/or pain, and the use of non-pharmacological measures to prevent or treat delirium, excluding data from centers 7 and 8 that only recruited patients at assessment point 1.

| **Variable** | **Delirium assessed with validated screening tool** | | **Sedation assessed with validated tool** | | **Pain assessed with validated tool** | | **Delirium, sedation, and pain assessed** | | **Non-pharmacological measures to prevent or treat delirium used ^a^** | |
| --- | --- | --- | --- | --- | --- | --- | --- | --- | --- | --- |
|  | **Odds ratio [95% CI]** | ***p*** | **Odds ratio [95% CI]** | ***p*** | **Odds ratio [95% CI]** | ***p*** | **Odds ratio [95% CI]** | ***p*** | **Odds ratio [95% CI]** | ***p*** |
| Gender, male | 0.621 [0.388; 0.992] | **0.046** | 1.231 [0.599; 2.530] | 0.572 | 0.658 [0.277; 1.563] | 0.343 | 0.821 [0.489; 1.377] | 0.454 | 0.738 [0.430; 1.267] | 0.27 |
| Age, yrs | 0.997 [0.983; 1.011] | 0.69 | 1.002 [0.980; 1.024] | 0.895 | 0.976 [0.947; 1.007] | 0.126 | 0.996 [0.980; 1.012] | 0.62 | 0.996 [0.979; 1.013] | 0.626 |
| ECMO, yes | 0.693 [0.352; 1.365] | 0.289 | 6.083 [0.762; 48.545] | 0.088 | 0.608 [0.150; 2.458] | 0.485 | 1.207 [0.539; 2.702] | 0.648 | 3.793 [1.092; 13.182] | **0.036** |
| Mechanical ventilation, yes | 0.257 [0.140; 0.471] | **< 0.001** | 5.844 [2.832; 12.058] | **< 0.001** | 0.871 [0.311; 2.442] | 0.793 | 0.829 [0.449; 1.529] | 0.549 | 2.374 [1.352; 4.167] | **0.003** |
| Assessment point 1 (reference) | 1 | - | 1 | - | 1 | - | 1 | - | 1 | - |
| Assessment point 2 | 1.134 [0.683; 1.882] | 0.627 | 0.885 [0.412; 1.901] | 0.754 | 1.897 [0.794; 4.533] | 0.15 | 2.862 [1.632; 5.017] | **< 0.001** | 1.666 [0.946; 2.934] | 0.077 |
| Assessment point 3 | 2.563 [1.408; 4.666] | **0.002** | 4.284 [1.423; 12.900] | **0.010** | NA ^b^ | NA ^b^ | 10.722 [4.698; 24.5] | **< 0.001** | 6.904 [2.948; 16.166] | **< 0.001** |
| Constant | 11.550 [3.091; 43.2] | **< 0.001** | 4.065 [0.320; 51.6] | 0.279 | 160.582 [7.074; 3645] | **0.001** | 2.430 [0.483; 12.2] | 0.282 | 2.132 [0.472; 9.641] | 0.325 |

To account for the clustered data, a random intercept for the treating country was included in the regression models. ^a^ Non-pharmacological measures to prevent or treat delirium comprised reorientation, early mobilization, and/or sensory shielding. ^b^ At assessment point 3, 104/106 (98%) of patients received a pain assessment. Hence, no OR could be estimated. ECMO, extracorporeal membrane oxygenation.

**Table S4.** Characteristics of delirium, pain, and sedation assessments, excluding data from centers 5, 6, 7, and 8 that did not recruit patients at all assessment points.

| **Variable** | **Assessment point** | | | ***p* ^a^** |
| --- | --- | --- | --- | --- |
|  | **1**  **(n = 113)** | **2**  **(n = 83)** | **3**  **(n = 106)** |  |
| Delirium screening with validated tool on assessment day | 81 (72 %) | 64 (77 %) | 82 (77 %) | 0.556 |
| CAM-ICU used | 81 (72 %) | 64 (77 %) | 82 (77 %) | 0.556 |
| Nu-DESC used | 0 (0 %) | 0 (0 %) | 0 (0 %) | - |
| ICDSC used | 0 (0 %) | 0 (0 %) | 0 (0 %) | - |
| Positive delirium screening,  n (% of those screened) | 14 (17 %) | 18 (28 %) | 11 (13 %) | 0.071 |
| Sedation depth assessed with validated tool on assessment day | 94 (83 %) | 68 (82 %) | 99 (93 %) | **0.033** |
| RASS used | 92 (81 %) | 68 (82 %) | 78 (74 %) | 0.263 |
| SAS used | 0 (0 %) | 0 (0 %) | 0 (0 %) | - |
| Other sedation scale used | 2 (2 %) | 0 (0 %) | 21 (20 %) ^b^ | **< 0.001** |
| Pain assessed with validated tool on assessment day | 101 (89 %) | 81 (98 %) | 104 (98 %) | **0.006** |
| VAS used | 6 (5 %) | 4 (5 %) | 4 (4 %) | 0.860 |
| NRS used | 53 (47 %) | 32 (39 %) | 54 (51 %) | 0.231 |
| BPS used | 34 (30 %) | 29 (35 %) | 42 (40 %) | 0.334 |
| BPS-NI used | 5 (4 %) | 5 (6 %) | 1 (1 %) | 0.154 |
| CPOT used | 0 (0 %) | 0 (0 %) | 0 (0 %) | - |
| Delirium, sedation, and pain assessed on assessment day | 66 (58 %) | 51 (61 %) | 77 (73 %) | 0.074 |
| Non-pharmacological measures to prevent or treat delirium | 57 (50 %) | 64 (77 %) | 96 (91 %) | **< 0.001** |
| Sensory shielding | 24 (21 %) | 22 (27 %) | 33 (31 %) | 0.249 |
| Reorientation (e.g. clock or whiteboard) | 35 (31 %) | 55 (66 %) | 71 (67 %) | **< 0.001** |
| Early mobilization | 37 (33 %) | 31 (37 %) | 61 (58 %) | **0.001** |

n (% of patients at assessment point) if not indicated otherwise. 6 centers participated at assessment points 1, 2, and 3. ^a^ Pearson’s χ² test if not indicated otherwise. ^b^ The AVPU (alert, verbal, pain, unresponsive) scale was applied in 21 patients in one UK center at assessment point 3. CAM-ICU, Confusion Assessment Method for the Intensive Care Unit; BPS, Behavioral Pain Scale; BPS-NI, Behavioral Pain Scale – Non-Intubated; CPOT, Critical Care Pain Observation Tool; ICDSC, Intensive Care Delirium Screening Checklist; NRS, Numeric Rating Scale; Nu-DESC Nursing Delirium Screening Scale; SAS, Riker Sedation Agitation Scale; RASS, Richmond Agitation Sedation Scale; VAS, Visual Analogue Scale.

**Table S5.** Multivariable mixed-effects logistic regression on the assessment of delirium, sedation, and/or pain, and the use of non-pharmacological measures to prevent or treat delirium, excluding data from centers 5, 6, 7, and 8 that did not recruit patients at all assessment points.

| **Variable** | **Delirium assessed with validated screening tool** | | **Sedation assessed with validated tool** | | **Pain assessed with validated tool** | | **Delirium, sedation, and pain assessed** | | **Non-pharmacological measures to prevent or treat delirium used ^a^** | |
| --- | --- | --- | --- | --- | --- | --- | --- | --- | --- | --- |
|  | **Odds ratio [95% CI]** | ***p*** | **Odds ratio [95% CI]** | ***p*** | **Odds ratio [95% CI]** | ***p*** | **Odds ratio [95% CI]** | ***p*** | **Odds ratio [95% CI]** | ***p*** |
| Gender, male | 0.625 [0.352; 1.109] | 0.108 | 0.802 [0.335; 1.919] | 0.62 | 1.541 [0.468; 5.071] | 0.477 | 0.824 [0.450; 1.509] | 0.531 | 0.529 [0.289; 0.970] | **0.039** |
| Age, yrs | 1.004 [0.987; 1.021] | 0.662 | 1.006 [0.979; 1.033] | 0.673 | 0.988 [0.948; 1.029] | 0.564 | 1.002 [0.984; 1.021] | 0.794 | 0.993 [0.974; 1.012] | 0.474 |
| ECMO, yes | 1.873 [0.566; 6.196] | 0.304 | NA ^b^ | NA ^b^ | 1.091 [0.092; 12.945] | 0.945 | 1.245 [0.345; 4.492] | 0.738 | 2.487 [0.471; 13.138] | 0.283 |
| Mechanical ventilation, yes | 0.199 [0.095; 0.417] | **< 0.001** | 5.275 [2.223; 12.519] | **< 0.001** | 0.652 [0.148; 2.876] | 0.572 | 0.438 [0.213; 0.899] | **0.024** | 2.008 [1.024; 3.938] | **0.042** |
| Assessment point 1 (reference) | 1 | - | 1 | - | 1 | - | 1 | - | 1 | - |
| Assessment point 2 | 1.203 [0.599; 2.415] | 0.604 | 0.973 [0.369; 2.562] | 0.956 | 4.874 [1.007; 23.583] | **0.049** | 3.337 [1.687; 6.600] | **0.001** | 2.469 [1.280; 4.762] | **0.007** |
| Assessment point 3 | 1.801 [0.936; 3.467] | 0.078 | 6.159 [1.913; 19.831] | **0.002** | NA ^c^ | NA ^c^ | 14.53 [6.253; 33.77] | **< 0.001** | 10.834 [4.526; 25.930] | **< 0.001** |
| Constant | 12.395 [2.59; 59.3] | **0.002** | 9.244 [0.368; 232] | 0.176 | 21.927 [0.599; 802] | 0.093 | 1.906 [0.358; 10.13] | 0.449 | 3.261 [0.615; 17.3] | 0.165 |

To account for the clustered data, a random intercept for the treating country was included in the regression models. ^a^ Non-pharmacological measures to prevent or treat delirium comprised reorientation, early mobilization, and/or sensory shielding. ^b^ All patients with ECMO received a sedation assessment. Hence, no OR could be estimated. ^c^ At assessment point 3, 104/106 (98%) of patients received a pain assessment. Hence, no OR could be estimated. ECMO, extracorporeal membrane oxygenation.

**Figure S1.** Share of patients that received an assessment for **(A)** delirium, **(B)** pain, **(C)** sedation, or **(D)** delirium, pain, and sedation, by assessment point and by center.

Comparing the first and third assessment point for the 6 centers contributing to all three assessment points, 4 improved their delirium assessment rate (2 worsened), 3 improved their pain assessment rate (1 stayed the same, 2 worsened), 1 improved their sedation assessment rate (4 stayed the same, 1 worsened), and 4 improved their PAD asse ssment rate (2 worsened). PAD, pain, agitation, and delirium.

**Figure S2.** Percentage of patients who received a delirium screening, sedation assessment, and/or pain assessment in **(A)** assessment point 1, **(B)** assessment point 2, or **(C)** assessment point 3.

Delirium: patient was screened for delirium. Sedation: patient received a sedation assessment. Pain: patient received a pain assessment. Assessment point 1 (n = 195), assessment point 2 (n = 129), assessment point 3 (n = 106). Due to rounding, percentages in assessment period 3 add up to 99%.
